# Simulation of anaerobic digestion processes using stochastic algorithm

**DOI:** 10.1186/s40201-014-0121-7

**Published:** 2014-09-04

**Authors:** Jegathambal Palanichamy, Sundarambal Palani

**Affiliations:** Water Institute, Karunya University, Coimbatore, Tamil Nadu 641114 India; Tropical Marine Science Institute, National University of Singapore, Singapore, 119227 Singapore

**Keywords:** Anaerobic digestion, Stochastic algorithm, Gillespie algorithm, Modeling

## Abstract

**Background:**

The Anaerobic Digestion (AD) processes involve numerous complex biological and chemical reactions occurring simultaneously. Appropriate and efficient models are to be developed for simulation of anaerobic digestion systems. Although several models have been developed, mostly they suffer from lack of knowledge on constants, complexity and weak generalization. The basis of the deterministic approach for modelling the physico and bio-chemical reactions occurring in the AD system is the law of mass action, which gives the simple relationship between the reaction rates and the species concentrations. The assumptions made in the deterministic models are not hold true for the reactions involving chemical species of low concentration. The stochastic behaviour of the physicochemical processes can be modeled at mesoscopic level by application of the stochastic algorithms.

**Method:**

In this paper a stochastic algorithm (Gillespie Tau Leap Method) developed in MATLAB was applied to predict the concentration of glucose, acids and methane formation at different time intervals. By this the performance of the digester system can be controlled. The processes given by ADM1 (Anaerobic Digestion Model 1) were taken for verification of the model.

**Results:**

The proposed model was verified by comparing the results of Gillespie’s algorithms with the deterministic solution for conversion of glucose into methane through degraders. At higher value of ‘τ‘ (timestep), the computational time required for reaching the steady state is more since the number of chosen reactions is less. When the simulation time step is reduced, the results are similar to ODE solver.

**Conclusion:**

It was concluded that the stochastic algorithm is a suitable approach for the simulation of complex anaerobic digestion processes. The accuracy of the results depends on the optimum selection of tau value.

## Background

Anaerobic Digestion (AD) is the process by which the complex form of organic matter such as carbohydrates, fats and proteins are converted into simpler form by the cells of microorganisms in the absence of oxygen. Energy production, high organic loading and low sludge production are major advantages of AD process. The energy produced can replace fossil fuel use, and also has positive effect on reduction of global warming. Modeling is a powerful tool which can be applied to simulate various processes occurring in the digester. Models are applied for parameter estimation also. Using the simulation results it is easy to predict and avoid digester failure. The modeling results give useful guidelines for the design of the digester also. The reaction system in an anaerobic digester is complex with many sequential and parallel steps. The reactions can be Biochemical or Physico – chemical in nature which involves species of higher and lower concentrations. A stochastic approach can be applied to simulate these reactions in exact manner. The complex organic matter which is called substrate is converted into simpler form through various steps by living cells called biomass. These cells grow at suitable environmental conditions of pH, temperature etc. They interact with the environment and substrates in a complicated way. Generally the biochemical processes include acidogenesis, acteogenesis, and anaerobic oxidation of Volatile Fatty acids, methanogenesis and extracellular hydrolysis step. The reaction kinetics of growth and decay of biomass and conversion of substrates from one form to another are detailed in the following sections.

### Modeling of anaerobic digestion

**Figure 1:**
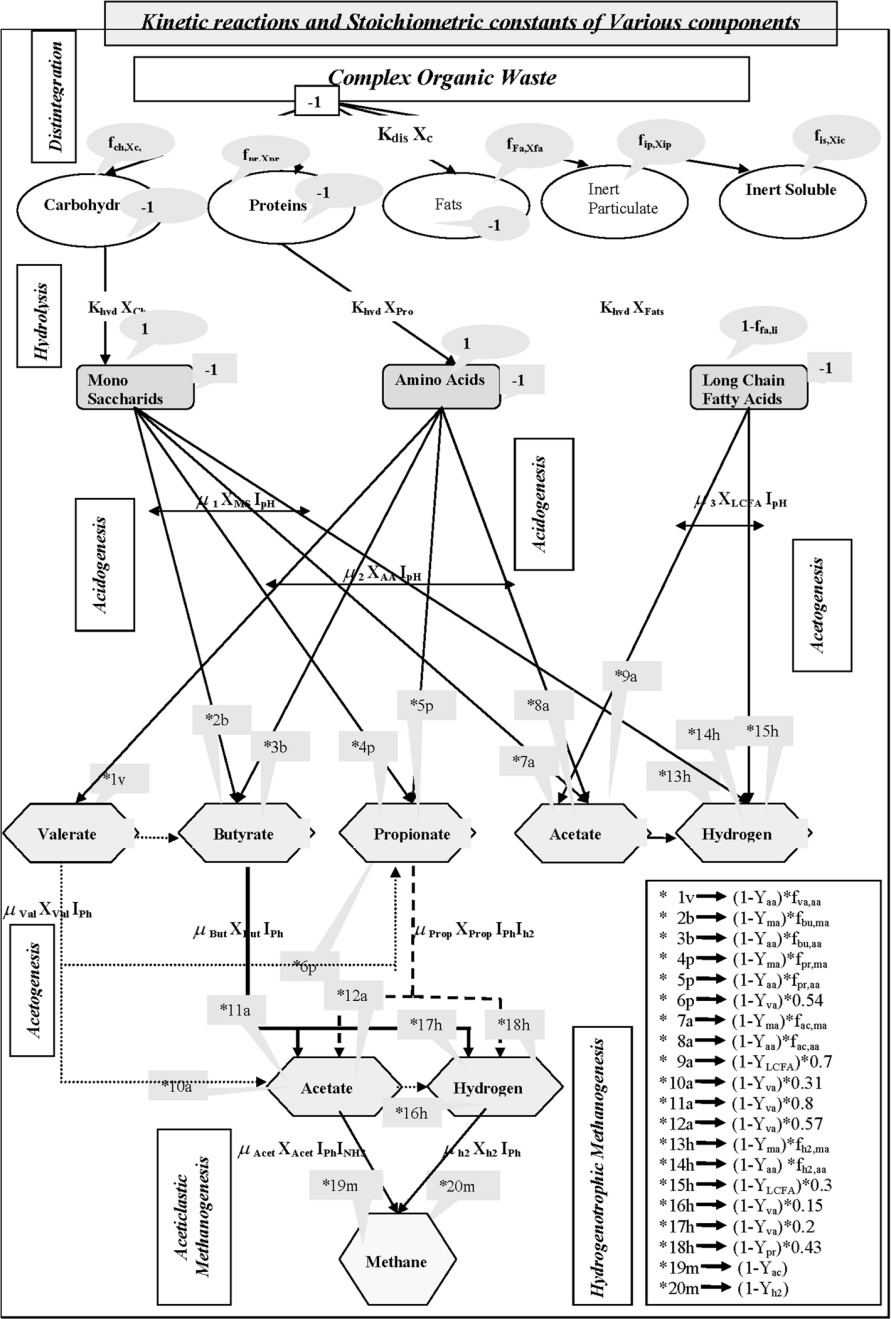
**Flowchart of Biochemical pathway in the anaerobic digestion system.**

Anaerobic digestion modeling was started in the early 1970’s when efficient operation of anaerobic systems was needed. The first developed models were simple with limited number of reaction equations [1,2]. Importance was given to simulation of final stage of the anaerobic digestion, methanogenesis [1–3]. Also attention was paid to the modeling of anaerobic digestion of “synthetic substrates” such as glucose [4]. Later, more complicated models of anaerobic digestion process of real and complex wastewater describing various bacterial groups, inhibition kinetics, pH calculations and gas dynamics were developed [5,6]. Although there is no common or unified modeling frame work for the anaerobic digestion process, ADM1 (Anaerobic Digestion Model 1) has been formulated by an international anaerobic modeling task group which was established in Sendai, Japan in 1997, and formally endorsed by IWA in 1998 [6]. Anaerobic digestion model no. 1 (ADM1) consists of 19 processes, 24 components and 56 relative stoichiometric and kinetic parameters. This paper focuses on simulation of five processes with 20 kinetic and stoichiometric parameters. In this work, four anaerobic microbial groups are considered for degradation: (i) glucose fermenting acidogens, (ii) propionic acid degrading acetogens, (iii) butyric acid degrading acetogens, acetoclatic methanogens and (iv) hydrogenotrohic methanogens. The flowchart of the degradation pathway of anaerobic processes is given in Figure 1.

Various models have been developed based on five major categories: models considering: (a) non-ionised Volatile Fatty Acids and total VFA inhibition; (b) H_2_ as regulator for VFA production; (c) ammonia inhibition. The growth of methanogenic population is greatly affected by un-dissociated acetic acid, un-ionised VFA and total VFA which cause a drop in pH. Several models have been developed taking the substrate (un-dissociated acetate and VFA) as inhibition factor [7,8]. The factor which regulates the amount of fatty acids generation is the liquid phase redox potential which is expressed as a function of H_2_ partial pressure. Due to sudden increase in organic loading, the accumulation of VFA takes place, since acetogens grow at a slower rate than the acidogens. This will increase pH which in turn the H_2_ partial pressure is increased. This will cause further accumulation of acids and thus methane generation is reduced. Few models have been developed based on the H_2_ partial pressure as inhibition factor [9–12] and manure as substrate and generated ammonia as inhibition factor [13–15]. Ammonia inhibits the methanogenesis process, thus acetic acid is accumulated. This in turn inhibits acetogenesis process and thus the total VFA accumulates. The reduction in pH causes decrease in ammonia concentration and the inhibition of methanogenesis process. Thus the ammonia inhibition is a self-regulatory.

In all the above models, many species are involved in more than one reaction. The reaction kinetics is solved by formulated Ordinary Differential Equations (ODE). To determine the rate of concentration of a particular species in time, all the reactions in which the species is involved are to be included during the formulation of ODEs. The complexity increases with increase in number of reactions and species. To avoid this, Stochastic Algorithm has been applied where the state of the system is updated based on the current state of the system and the transition probability.

### Kinetics of anaerobic digestion

The complex organic matter which is called substrate is converted into simpler form through various steps by living cells called biomass. These cells grow at suitable environmental conditions of pH, temperature etc. They interact with the environment and substrates in a complicated way. The reaction kinetics of growth and decay of biomass and conversion of substrates from one form to another are detailed in the following sections.

### Kinetics of biomass growth and decay

Once the inoculum is introduced into anaerobic digester, the cells pass through lag phase where they adjust to the new environment. Once they get accustomed to the new environment, they start growing in exponential phase called log phase. During this phase, the specific growth rate remains constant. The specific growth rate is given by dx/dt = µx. When the growth limiting substrate is exhausted, the growth remains stationary and reaches the maintenance mode in the stationary phase. When there is no substrate, the cell population slowly starts decreasing in the death phase. In each growing culture, there is a maximum rate of growth per unit biomass with unlimited substrate in the given environment (μ_max_).

### Kinetic model of biomass growth

A kinetic model represents the cell population kinetics. The models can be unstructured, structured unsegregated and segregated. In the unstructured model a single substrate is considered as growth limiting one. Multiple substrates are considered in structured model. In unsegregated model, the average properties of the cell population are considered. In segregated model the discrete and heterogeneity of cell populations are considered. Also various kinetic models such as Maltha’s Law, Slater model and Monod model are used in modeling AD processes. In Maltha’s law the rate of increase of biomass is a function of microbial population only (F(X) = µx). In this model, lag or death phase is not considered. It is assumed that there is unrestricted growth of biomass. In Slater model, final population of biomass is also included. This is represented by logistic equation of biomass growth which relates the specific growth rate μ, biomass concentration X, maximum specific growth rate μ_max_ and final population X_f_ . µ = µ_max_(1 ‐ X/X_f_). This is an empirical formula which can be applied in batch studies. Generally, Monod kinetic model is applied in most of the biological wastewater treatment processes. In this model, when one of the substrate concentrations (S) is Limiting, the biomass growth is represented by µ = µ_max_S/(K_s_ + S). K_s_ is the value of the limiting nutrient concentration at which the specific growth rate is half its maximum value.

### Advantage and limitation of Monod equation

In this model, the kinetic parameters (µ_max ,_K_s_) which describe the microbial processes are able to predict the conditions of maximum growth and when the activity will cease. The main disadvantage of this model is that since the kinetic parameters (µ_max ,_K_s_) vary with substrate, one set of parameters cannot describe biological process at short and longer retention time. To overcome this limitation, first – order models are used [16].

### Application of Stochastic algorithm for simulation of kinetic reactions in the anaerobic digestor

In this work, four anaerobic microbial groups are considered for degradation: (i) glucose fermenting acidogens; (ii) propionic acid degrading acetogens; (iii) butyric acid degrading actetogens, acetoclatic methanogens and (iv) hydrogenotrohic methanogens.

### Step I: Acidogenesis

After hydrolysis of complex substrate into simpler organic compounds such as glucose, short chain fatty organic matter takes place, acidogens degrade glucose into acetic, propionic and butyric acids. Hydrogen is considered as an inhibitor for acidogens according to Embden- Myerhoof pathway since the correlation of NAD^+^/NADH in the cells of biomass depend on the concentration of hydrogen.

### Step II: Acetogenesis

In this slowly growing and pH sensitive acteogens oxidize propionic and butyric acids to acetate. At high partial pressure of hydrogen, it acts as an inhibitor in acetogenesis phase. Also acetate inhibition of the propionic and butyric acid degradation step has been considered in numerous studies. So this can be represented by non-competitive type inhibition model.

### Step III – Acetoclastic methanogenic stage

In this step, pH –sensitive and slowly growing acetocalstic methanogens reduce acetate to methane. Here, free ammonia is the inhibitor for the growth of methanogens. The stoichiometric equation and specific growth kinetic reactions are given below:

### Step IV – Hydrogenotrohic methanogenesis

There may be growth limitations due to deficiency of CO_2_ in the reaction system due to digestion of propionic and butyric acids by acetogens. So dual substrate form of monod equation can be applied to represent the specific growth rate of Hydrogenotrohic methanogens.

### Biochemical reactions and their kinetics in the anaerobic digestion system

Biochemical reactions and their kinetics in the anaerobic digestion system were assumed to follow first order reactions (hydrolysis), monod type kinetic reactions and inhibition reaction. The complex particulate waste from industries or household is first disintegrated into carbohydrate, protein and lip (both particulate and soluble inert material). During hydrolysis by extra cellular enzymes (hydrolases), monossaccharides, amino acids and Long Chain Fatty Acids (LCFA) are formed. All these bio-chemical extracellular steps were assumed as first order [6]. The first order kinetic model is an empirical relation, which assumes that the hydrolysis rate is a linear function of the available biodegradable substrate at a certain pH and temperature. The acidogenic bacteria turn the products of hydrolysis into simple organic compounds such as short chain Volatile Acids (VA), e.g. propionic, formic, lactic, and butyric and alcohols such as ethanol, methanol, glycerol and acetone. Then two types of acetogenic mechanism can occur [5] (a) acetogenic hydrogenations and (b) acetogenic dehydrogenations. In acetogenic hydrogenations, the organic acids formed are subsequently converted by acetogenic bacteria to acetate as the main product. Acetogenic dehydrogenations include the anaerobic oxidation of volatile long and short chain fatty acids. In this reaction, acetate is formed from the separated carbon atoms. During this process, due to high hydrogen partial pressure, oxidation process can be inhibited. So the hydrogen produced by these organisms is consumed by a hydrogen-utilizing methanogenic group and acetate by an aceticlastic methanogenic group. Almost the 64–70% of methane production is from acetate. Methanogenic bacteria are very sensitive to pH, temperature, loading rate and other compounds. All the substrate uptake reactions are intracellular reactions and they are modeled using Monod kinetics reaction (single Monod, double Monod and also with competitive and non competitive reactions) Methane is considered to be water insoluble, whereas the carbondioxide is partially soluble and partly escapes into gas phase. When temporary accumulation of Volatile fatty acids occurs, the pH of the digester is reduced. This will increase the concentration of unionized VFA in the system. This will inhibit methanogenic activity. Inhibition function includes pH, hydrogen and free ammonia. Hydrogen and free ammonia inhibition can be represented by non-competitive reaction whereas pH inhibition can be represented by empirical equations. The inorganic nitrogen uptake is represented by competitive secondary Monod-kinetics, where the prevention of growth due to limitation of nitrogen and competition for uptake of butyrate and valerate occur.

### Deterministic and Stochastic approaches for reaction simulation

In the deterministic approach for reaction simulation, the time evolution of the system is considered as continuous which is governed by a set of coupled ODEs. But the stochastic approach regards the time evolution similar to a random walk process which is governed by a single differential equation which is called the master equation. In the deterministic approach, given the initial concentration of various species in a free homogeneous macroscopic environment, the concentration at all future time intervals is determined by averaging random fluctuations to produce a predictable deterministic behaviour [17]. All the elementary reactions (first, second order) follow the reaction rate law where the rate of the reaction is always proportional to the concentration of each reactant involved in the reaction. The ODE solvers propagate the system’s state using finite time steps. For non-linear reactions, extremely small time steps need to be adopted to keep the numerical exactness. In that case adaptive step size or implicit method is recommendable. The ODE approach is empirically accurate for reaction systems where large concentrations occur and may not be adequate for systems with small concentrations. In the deterministic approach, the species population is described by a continuous state although a chemical reaction involves random collisions between individual species. Also a predictable system is assumed for the reaction rate or velocity and time evolution. These two assumptions are not appropriate for a system with low concentration of species where high relative fluctuations due to stochastic effect occur. Stochasticity in the state change occurs as intrinsic and extrinsic stochasticity. The intrinsic stochasticity is inherent to the system and mainly arises due to low concentration of species and extrinsic stochasticity arises due to the random variation of environmental factors.

### Concept of master equation

**Figure 2:**
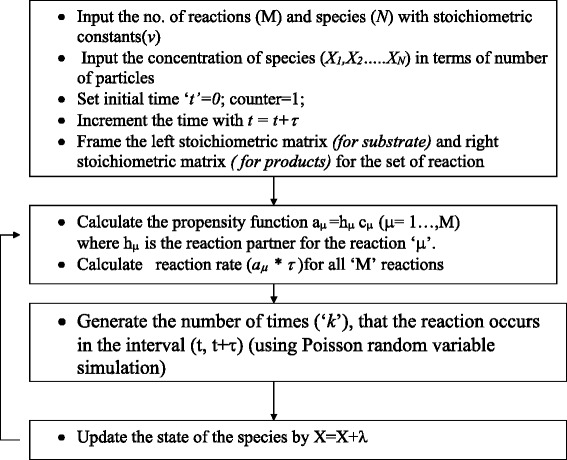
**Flowchart of Gillespie’s Tau Leap Method [** 22 **].**

Master equation describes the transition of a system from one state to another state using probabilistic formulations. The state of system is determined by incoming and outgoing transitions. Since the state change of a chemical reaction system occurs in a discrete number, the probability to find the current state of the system can be described according to the Master Equation approach. In the Master Equation for reactions, all states are represented by a discrete number of molecules and transition intensities are given by reactions per second. Various Stochastic Simulation Algorithms (SSA) such as Gillespie Direct Method, Tau leap methods, slow reaction method and adaptive tau leap method to generate the trajectories by evolving the reaction type and the time of occurrence of the reaction through the probability distribution [18–22]. It was developed based on the assumption that the reaction system is well mixed and homogeneous. In this work, an explicit Tau leap method is adopted for reaction simulation. In Gillespie’s Tau Leap Method, leaping occurs over sub time intervals τ. The number of reactions occurring in the subinterval is determined. The number of times *k*_*j*_ (τ; *X* (*t*),*t*) , that the reaction event *R*_μ_ occurs in time interval [t,t + τ ) is given by a Poisson random variable, P(*a*_*j*_ (*X* (*t*)),τ ), where *j* = 1,.. *M*. The flowchart of Gillespie’s Tau Leap Method is outlined in Figure 2. The key point of Gillespie’s Tau Leap Method lies in the selection of time step. ‘τ’ will be approaching to 1/*a*_0_(*X* (*t*)) when the number of reactions or the concentration of the reactant species is low. In that case it will be equivalent to Gillespie’s Direct Method where only one reaction is chosen in a time step. In these cases, the algorithm becomes inefficient.

### Application of a stochastic algorithm for the simulation of anaerobic digestion processes

**Figure 3:**
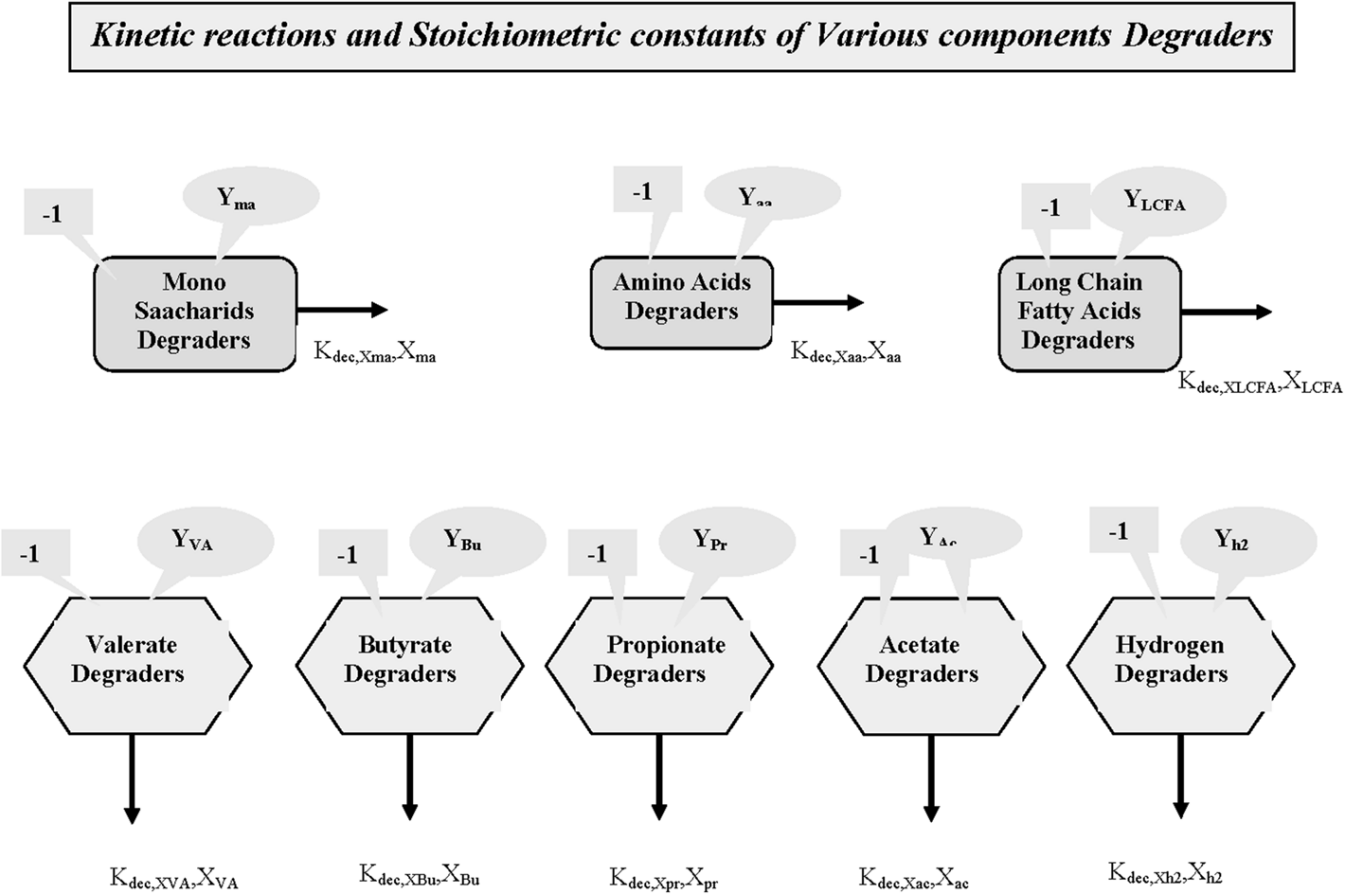
**Flowchart of processes involved with species degraders.**

Based on stoichiometric values, each species is apportioned with theoretical numbers of particles based on the assumed mass. The fluctuations which occur due to stochastic nature of simulation can become less pronounced either by repeating the simulation with less number of particles and computing the mean or by assuming more number of particles. According to the theoretical statistical physics, the fluctuations in the system are inversely proportional to the square root of the number of particles involved in the simulation. So in this work, more number of particles is assigned for a particular species in which the substrate Glucose is assigned with theoretical number of particles proportional to the concentration in the ratio of 1:5. Figure 3 shows Flowchart of processes involved with species degraders.

**Figure 4:**
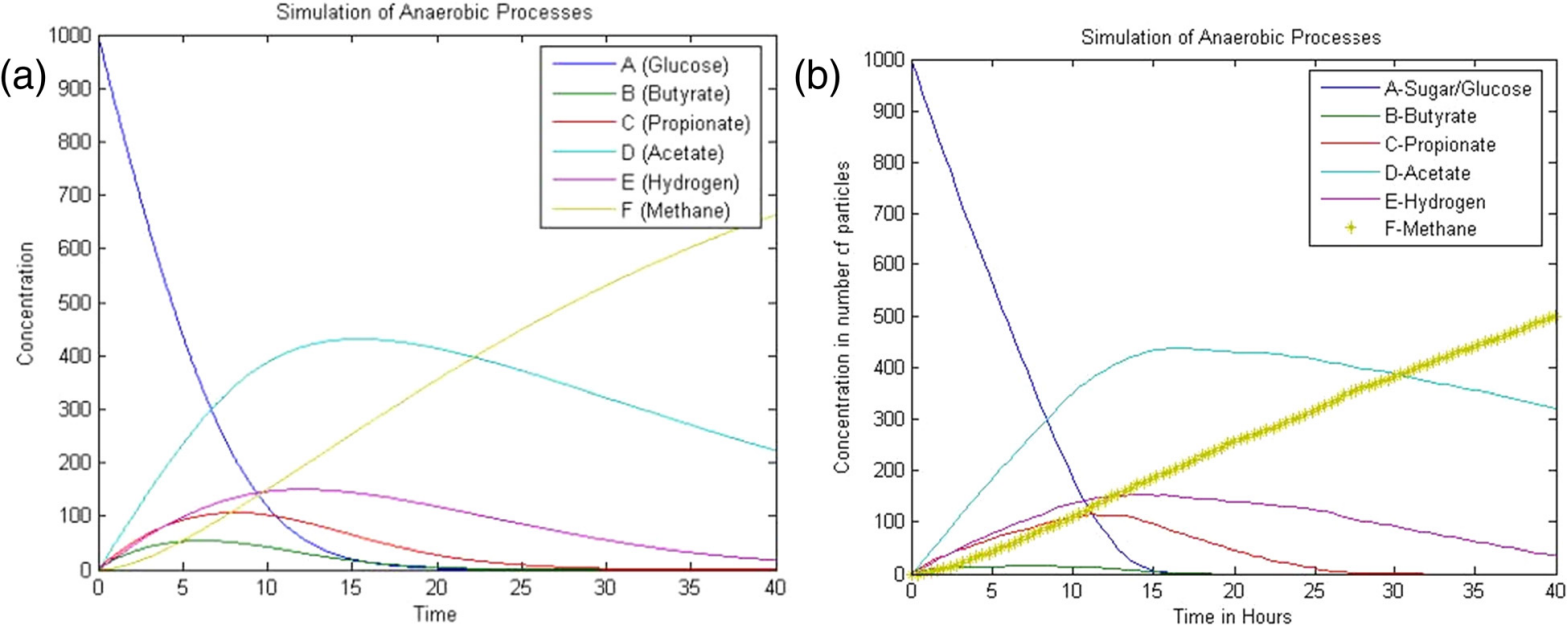
**The simulation results obtained from (a) SBTOOLBOX in Matlab and (b) Stochastic Algorithm.**

**Figure 5:**
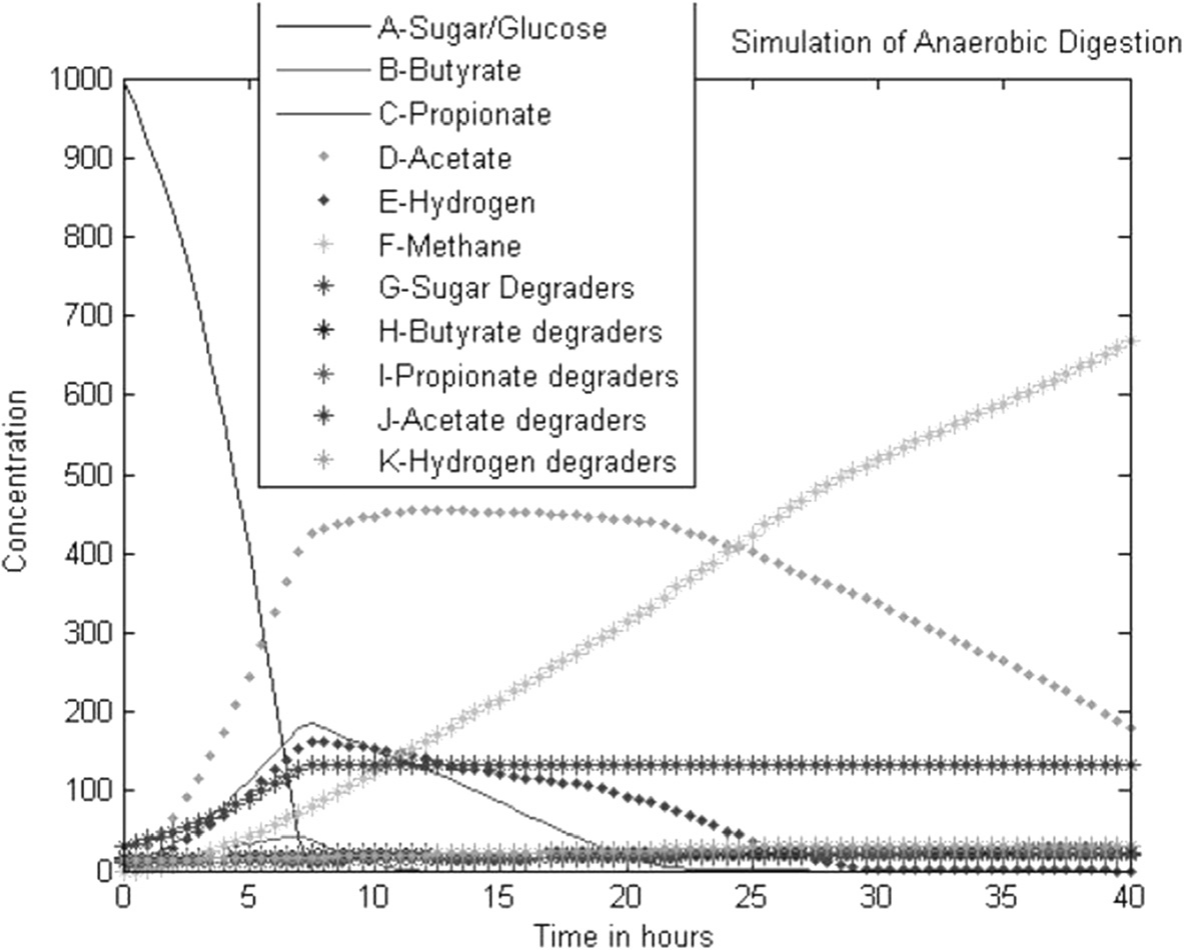
**Simulation results with change in the species degraders.**

The kinetic parameters in this model μ_max_,_glu_ = 1.25, μ_max_,_bu_ = 0.833, μ_max,pro_ = 0.542, μ_max,ace_ = 0.333, μ_max,hyd_ = 0.35, K_s_ = 500, K_b_ = 200, K_p_ = 100, K_a_ = 150, K_h_ = 150, where μ_maxS_ are maximum uptake rate of various species, K’s are the half saturation value [23]. The yield of product on the substrate (f) and the yield of biomass on substrate (Y) of the various reactions involved in biodegradation are given in the Table 1. Monod reactions are used as rate reactions to determine the next state of the system. The propensity function which is the rate of each reaction a_μ_ is determined. Then based on the total reaction rate of the system, a reaction is chosen by Poisson random variable. The time step is assumed in such a way that more than one reaction is chosen in a single simulation step. If higher value is chosen, more reactions are chosen where there will be great variation in the propensity function of the chosen reactions. Based on the chosen reactions in a given time step and the stoichiometric constants of products and substrates of the chosen reactions, the number of particles assigned for the reactant and product species involved in the chosen reactions will be modified. So the accuracy of the algorithm depends on the selection of the time step. The simulation results obtained from SBTOOLBOX in Matlab and stochastic algorithm s are shown in Figures 4 and 5.

**Table 1:** **Stoichiometric constants for substrates and products in the model**

**Sugar/Glucose**	**Constant**	**Constant**	**Constant**	**Constant**	**Constant**
*G*	10	0	0	0	0
*B*	0	1	0	0	0
*P*	0	0	1	0	0
*A*	0	0	0	1	0
*H*	0	0	0	0	1
*M*	0	0	0	0	0
*G#*	0	0	0	0	0
*B#*	0.117	0	0	0	0
*P#*	0.243	0	0	0	0
*A#*	0.369	0.752	0.5472	0	0
*H#*	0.171	0.188	0.4128	0	0
*M#*	0	0	0	0.95	0.94

Note: G; B, P, A, H, M are the stoichiometric constants for substrates (based on ‘f’ and ‘Y’ values); G#, B#; P#; A#, H#; M# are the stoichiometric constants for products (based on ‘f’ and ‘Y’ values). Sugar/Glucose; B. Butyrate; C. Propionate; D. Acetate; E. Hydrogen; F. Methane; G. Sugar degraders; H. Butyrate degraders; I. Propionate degraders J. Acetate degraders; K. Hydrogen degraders.

When simulation time step is more than 1, the number of selections of the chosen reaction is insignificant and the formation of methane is less. Update of sugar takes longer time to reach the steady state. The simulation of formation of acids and formation of methane are rapid. When the tau value is reduced, the simulation results approaches the results of simulation using ODE solver. This is due to the selection of reactions where the propensity function remains constant. At the time step of 0.4, the simulation results get closer to deterministic values. Thus the accuracy of simulation depends on the selection of the optimal value of tau.

## Conclusion

When more number of reactions are involved in a biochemical processes, the rate of change of concentration of species is determined by evolving a rate equation for each species. As the number of species increases, the number of rate of reactions also increases to evolve the change in the concentration of species. But it is a complex process. The difficulty in formulation of Ordinary Differential Equation (ODE) for reaction simulation can be overcome using Gillespie’s algorithms where the reactions can be represented in the form of matrix which involves the stoichiometric constants and the reaction rate constants. Stochastic Algorithm (SA) has been identified as a better solution where multiscale concentration species are involved in reactions such as sorption, precipitation, degradation, sequential and parallel decay reactions etc. In this paper, the feasibility of applying SA (Gillespie Tauleap Method) in simulation of various bio chemical processes occurring during anaerobic degradation of complex organic matter has been studied.
